# Nanoformulations to Enhance the Bioavailability and Physiological Functions of Polyphenols

**DOI:** 10.3390/molecules25204613

**Published:** 2020-10-10

**Authors:** Bingyan Yang, Yixin Dong, Fei Wang, Yu Zhang

**Affiliations:** Jiangsu Provincial Key Lab for the Chemistry and Utilization of Agro-Forest Biomass, Jiangsu Key Lab of Biomass-Based Green Fuels and Chemicals, Jiangsu Co-Innovation Center of Efficient Processing and Utilization of Forest Resources, College of Chemical Engineering, Nanjing Forestry University, Nanjing 210037, China; taylor1106@163.com (B.Y.); kaydong417@163.com (Y.D.); hgwf@njfu.edu.cn (F.W.)

**Keywords:** polyphenols, bioavailability, loading, nanoformulations

## Abstract

Polyphenols are micronutrients that are widely present in human daily diets. Numerous studies have demonstrated their potential as antioxidants and anti-inflammatory agents, and for cancer prevention, heart protection and the treatment of neurodegenerative diseases. However, due to their vulnerability to environmental conditions and low bioavailability, their application in the food and medical fields is greatly limited. Nanoformulations, as excellent drug delivery systems, can overcome these limitations and maximize the pharmacological effects of polyphenols. In this review, we summarize the biological activities of polyphenols, together with systems for their delivery, including phospholipid complexes, lipid-based nanoparticles, protein-based nanoparticles, niosomes, polymers, micelles, emulsions and metal nanoparticles. The application of polyphenol nanoparticles in food and medicine is also discussed. Although loading into nanoparticles solves the main limitation to application of polyphenolic compounds, there are some concerns about their toxicological safety after entry into the human body. It is therefore necessary to conduct toxicity studies and residue analysis on the carrier.

## 1. Introduction

Many effective medical treatments have originated from plant extracts, which are important sources of materials for the treatment of many diseases [1]. Phenolic compounds are widely present as secondary metabolites in all vascular plants [2,3,4,5]. They play important roles in the growth and development of plants, and are involved in defense against ultraviolet light and pathogens [6]. In recent years, because of their potential positive role in human metabolism, they have attracted increasing attention and research. These compounds have biological properties that include antioxidant, anti-inflammatory, antibacterial, anticancer and cardiovascular protection activities [7,8,9].

However, the use of phenolic compounds in humans is limited by many factors, such as low solubility, poor permeability, instability, rapid release, susceptibility to environmental influences and low bioavailability [10,11]. In order to overcome these limitations, polyphenols are often loaded into various carriers to enhance their bioavailability. This can increase biocompatibility, prevent degradation caused by the external environment, and prevent interaction with other components in the human body. Nanocarriers have been demonstrated to be excellent materials for encapsulating phenolic compounds and improving their bioavailability.

With the rapid development of nanotechnology in the food and pharmaceutical industries, many advanced nanoparticles have been developed to protect and control/target the release of biologically active ingredients, including various polyphenols [12,13,14,15]. The size of nanoparticles and nanocarriers is in the range of 1–100 nm [16]. By loading phenolic compounds into nanoparticles, not only can their bioavailability be improved, but also the controlled/targeted release and protection of active substances can be achieved [17]. In recent years, many nanoparticles have been developed for the delivery of polyphenolic compounds, including liposomes, phospholipid complexes, niosomes, protein-based nanoparticles, micelles, emulsions and metal nanoparticles.

**Figure 1:**
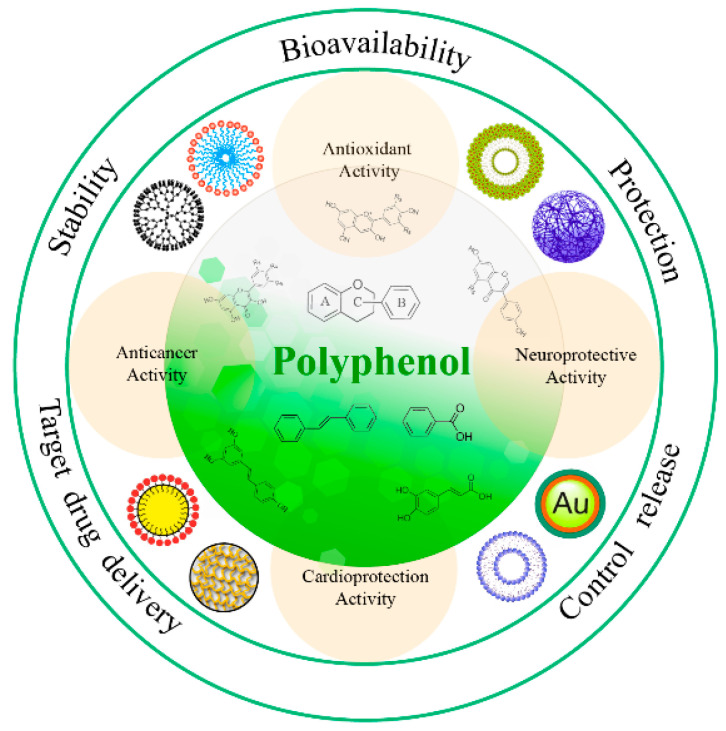
Schematic representation of nanoformulations to enhance the bioavailability and physiological functions of polyphenols.

This review first summarizes the biological activities of polyphenolic compounds, and then introduces the application of a variety of nanoparticles to improve their bioavailability (Figure 1).

## 2. Bioactivities of Polyphenols

With the continuous emergence of the benefits of polyphenols, there is growing interest in the study of its biological activities, such as antioxidant, heart protection, cancer prevention and nerve protection. Polyphenols play an irreplaceable role in medicine, food, health products and other fields.

### 2.1. Antioxidant Activity

Among all biological activities of phenolic compounds, antioxidant activity is one of its most important activities, and has been extensively studied, including scavenging free radicals, inhibiting the oxidation of lipids and reducing the formation of hydrogen peroxide, etc. Phenolic compounds have significant antioxidant properties, as the structure contains a large number of hydroxyl groups, which has a great influence on the ability to scavenge free radicals and chelate metal ions [18,19]. Polyphenols can neutralize free radicals by providing electrons or hydrogen atoms to a variety of reactive oxygen species (ROS). Acting as metal ion chelating agents, polyphenols can transform peroxides or metal oxides into stable substances and thus disrupt the proliferation stage of lipid autotrophic chain reactions [20,21]. Previous studies have shown that some flavonoids can directly scavenge peroxides, while others have the ability to scavenge a strong oxygen-derived free radical (such as peroxynitrite) to inhibit low-density lipoprotein (LDL) oxidation [22]. It is true that phenolic compounds are good antioxidants, however, when losing electrons or acting as reducing agents, the molecules themselves will be converted into free radicals, and the interactions with transition metal ions will also lead to the formation of pro-oxidants [23]. Therefore, it is necessary to understand correctly its activity and control its dosage reasonably.

The balance of the oxidative defense mechanism in human cells is maintained by the enzymatic redox system, which mainly includes catalase (CAT), hydrogen peroxide dismutase (SOD), glutathione peroxidase (GPx), glutathione reductase (GR) and peroxidase (PRXs) [24]. However, under the oxidative stress condition of excessive reactive oxygen, the defense ability will be greatly reduced [25]. It has been proved that polyphenols can restore equilibrium by increasing the activity of SOD, CAT, GPx, GR and PRXs, thus preventing systemic or local inflammation [26]. The antioxidant activity of plant polyphenol extracts has been extensively studied in different biological systems (Table 1).

### 2.2. Cardioprotection Activity

Cardiovascular disease (CVD), also known as circulatory disease, is a range of diseases that involve the circulatory system, including coronary artery disease, stroke, heart failure and high blood pressure. Numerous studies have shown that polyphenols have beneficial effects on cardiovascular health, and consuming foods rich in polyphenols can reduce the risk of these diseases [54,55,56,57].

Wang et al. [58] reported that anthocyanins, proanthocyanidins, flavonoids, and flavan-3-ols in polyphenols are closely related to the incidence of CVD. Increasing the daily intake of flavonols by 10 mg can reduce the risk of cardiovascular disease by 5%. The meta-analysis by Menezes et al. [59] also showed that the intake of flavonols reduced the risk of CVD, but the impact of flavonols intake on lipid levels may vary depending on the country and health status of individuals.

Tang et al. [60] demonstrated in a study on the risk of stroke that ingesting 100 mg of flavonoids per day reduced the stroke rate by approximately 10%. A similar study conducted by Wang et al. [61] demonstrated that an intake of 20 mg of flavonols per day was associated with a 14% reduction in stroke risk. Studies have shown that the main polyphenolic compounds that can significantly reduce the risk of hypertension are flavonoids [62,63,64]. Through a meta-analysis of randomized controlled trials, Huang and coworkers concluded that participants who took quercetin for 8 weeks or longer showed significant changes in HDL cholesterol and triglyceride levels [65]. Important evidence from epidemiological investigations suggests that dietary polyphenols may treat and prevent type 2 diabetes. Cao and coworkers have demonstrated that resveratrol can improve blood glucose control in subjects with insulin resistance, and anthocyanins can lower blood glucose or optimize insulin secretion and resistance [66]. It is reported that genistein could significantly improve glycemic control and sensitivity to insulin in postmenopausal women [67].

Atherosclerosis is a chronic inflammatory disease that occurs in vulnerable areas of middle arteries. It may have existed for many years before it develops and produces physiological states, such as acute myocardial infarction, unstable angina, or sudden cardiac death [68]. Anthocyanins can increase blood lipids, inhibit inflammation, and improve endothelium-dependent vasodilation by activating NO-cGMP signaling pathway [69]. It is also demonstrated that, by inhibiting the activity of phosphodiesterase-5, the blood vessels can be relaxed, thus reducing the risk of cardiovascular disease [70].

The formation of thrombosis is one of the main causes of myocardial infarction, ischemic heart disease and other diseases. Plenty of evidence has proven that dietary polyphenols can reduce the formation of thrombus [71,72]. Red wine, rich in polyphenols (such as resveratrol, proanthocyanidins, etc.), is known to have the function of protecting the heart. Extensive data have shown that resveratrol can prevent thrombosis by reducing oxidative stress and platelet aggregation, thereby protecting blood vessels [73,74].

### 2.3. Anticancer Activities

Cancer is a cell proliferative disease caused and controlled by genetic mutations, resulting from various factors, such as diet, radiation, unhealthy life habits (such as regular drinking and smoking) and infectious microorganisms [75]. Considerable evidence has proven that it is the plant secondary metabolites, polyphenols, that make a significant contribution to anticancer activity [76,77,78,79]. Polyphenols are mainly used to prevent cancer by inhibiting cancer cell activation, promotion, angiogenesis, and strengthening the immune system [80,81,82]. Polyphenols have preventive and therapeutic effects on many cancers, such as esophageal, gastric, colon, liver, lung, breast, ovarian and skin cancer (Table 2) [83,84,85].

Grosso et al. [86] conducted a systematic evaluation of the role of polyphenols in cancer prevention, and the analysis showed that isoflavones significantly reduced the risk of gastric, lung, breast and colorectal cancers, by directly inhibiting oxidative stress, oxidative damage, anti-angiogenesis and anti-metastasis, while total flavones had little effect on the risk of breast cancer. Another study reviewed the role of flavonoids in colon cancer risk and found that the sustained consumption of flavonoids appeared to be an effective complementary treatment that reduced colon cancer risk from cellular antioxidants and anti-inflammatory effects [87]. The study also revealed that the flavonoid subclasses had a significant synergistic effect on preventing tumorigenesis, tumor growth and promoting apoptosis of cancer cells [87]. Similar studies by Chang and coworkers proved that the intake of quercetin and other flavonol compounds could reduce the risk of colon cancer, while the intake of apigenin and other flavonoids could reduce the risk of rectal cancer [88].

**Table 2:** Anti-cancer effects of some polyphenols.

Polyphenol	Cancer Type/ Cell Line	Major Outcomes	References
Curcumin	MBA-MB-231cells, MCF-7 cells	Down-regulate the mRNA expression of Vimentin, Fibronectin, and β-catenin; up-regulate E-cadherin mRNA expression levels	[89]
	HCT-116 cells	Reduce the expression of SIRT1 protein, suppress the oncogenicity of human-colon cancer cells	[90]
	T98G, U87MG, T67 cells, HCT-116 cells	Inhibit AP-1 and NF-κB signaling pathways, suppress JNK activation induced by carcinogens	[91]
Resveratrol	LNCaP cells	Induce the expression of COX-2, promoting ERK1/2 activation, and facilitate p53-dependent anti-proliferation gene expression	[92]
	NSCLC cells	Prevent tumorigenesis and progression, and down-regulate EGFR/Akt/ERK1/2 signaling pathway	[93]
	Apc10.1 cells	Show superior efficacy than high doses due to the pro-oxidant activity and AMPK signaling upregulation	[94]
	Hela cells	Inhibit the expression of PLSCR1, leading to the growth inhibition of HeLa cells	[95]
	SGC7901, BGC823 cells	Inhibit the invasion and migration of human gastric cancer cells	[96]
Quercetin	MDA-MB-231 cells	Increase FasL mRNA expression and p51, p21, and GADD45 signaling activities, induce protein level, transcriptional activity, and nuclear translocation of Foxo3a	[97]
	AsPC-1, CRL-4023, PANC-1 cells	Reduce the expression levels of cellular FLICE-like inhibitory protein, activate c-Jun N-terminal kinase (JNK)	[98]
	A549 cells	Trigger BCL2/BAX-mediated apoptosis, as well as necrosis and mitotic catastrophe	[99]
	PC-3 cells	Decrease tumor improvement, down-regulate Ki67, and enhance caspase 7	[100]
EGCG	Breast T47D	Up-regulate PTEN, CASP3, CASP9, down-regulate AKT	[101]
Genistein	Pancreatic Mia-PaCa2	Induce mitochondrial apoptosis, block cell cycle and regulate STAT3	[102]
	Colorectal HCT 116	Inhibite cell proliferation, induce apoptosis of colorectal cancer cells	[103]
Daidzein	Colorectal HT-29, MIA PaCa-2	Cytotoxic effects on both MIA PaCa-2 and HT-29 cell lines	[104]
	Ovarian SKOV3	Up-regulate B-cell lymphoma 2-associated X protein, cytochrome c, down-regulate pCdc25c, Cdc25c	[105]
	BEL-7402	Increased the levels of reactive oxygen species (ROS) and induce a decrease in mitochondrial membrane potential	[106]
Chrysin	HCT-116; HepG2; Hep 3B	The combination of chrysin and cisplatin promoted apoptosis of HepG2 cells in both dose- and time- dependent manners	[107]
	A549	Reinforce the therapeutic efficacy of DTX and mitigate edema	[108]

### 2.4. Neuroprotective Activity

With the continuous improvement of medical standards, the average life span of humans has been significantly extended, but correspondingly, diseases related to brain aging caused by an aging population have also increased significantly, such as cognitive and neurodegenerative diseases [109]. Neurodegenerative diseases, characterized by the progressive loss of functions of a large number of neurons and neural stem cells, leading to sensory deficits and cognitive impairments, are a type of progressive, disabling and severely fatal complex disease [110]. Studies have found that all neurodegenerative diseases share a common cellular and molecular mechanism, that is, oxidative stress accumulation, inflammation, protein misfolding and aggregation, neurotoxicity, etc. [111,112,113].

In addition to genetic and environmental factors, the increase of oxidative stress in cells is considered to be the main cause of neurodegenerative diseases [114,115]. A large number of studies have shown that polyphenols can inhibit the increase of oxidative stress through many mechanisms. For example, polyphenols can enhance the activity of detoxification and antioxidant enzymes by activating the Nrf2 pathway [116,117,118]. They can also regulate the activity of reactive oxygen generation enzymes and modify the structural integrity and metabolic efficiency of mitochondria [119,120]. Inflammation is also the cause of such diseases. Polyphenols can regulate the expression of pro-inflammatory genes such as nitric oxide, lipoxygenase, cyclooxygenase (COX) and chemokines [115,121]. In addition, the neuroprotective effect of polyphenol compounds is also attributed to the reduction of amyloid aggregation and/or the formation of precursors. Curcumin has been shown to have anti-amyloidosis activity, and can not only inhibit the formation of new β-aggregates, but also decompose already formed aggregates [122]. Pomegranate polyphenols, myricetin, luteolin and honokiol variably altered the morphology of Aβ aggregation, the flavonoids all bound in a similar hydrophobic region of the amyloid pentamer and exhibited the most obvious inhibitory effect on Aβ_1-42_ aggregation [123].

## 3. Nanoformulations for Loading and Delivery of Polyphenols

As mentioned earlier, polyphenols have been widely concerned and applied in many fields due to various beneficial functions, but some of their restrictive factors have greatly hindered their applications, in vivo and in clinic. These factors mainly include low solubility, permeability, and bioavailability. In order to overcome the limitations, nanocarriers have been developed extensively [124]. The unique physicochemical properties of nanocarriers, such as high loading, drug protection and tumor cell penetration, provide preconditions for the delivery of polyphenols and other drugs [125,126]. At present, the delivery systems like phytosomes, liposomes, niosomes, protein-based nanoparticles, polymer nanoparticles, microspheres and emulsions have emerged as attractive options for controlled bioactive systems [127,128].

### 3.1. Phytosome

**Figure 2:**
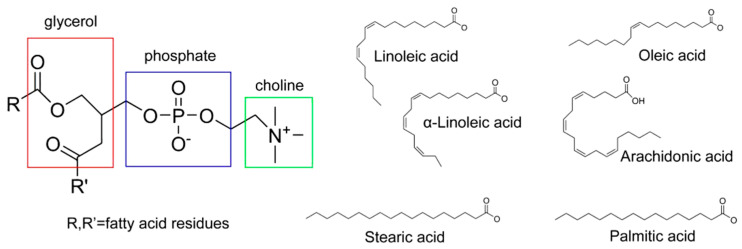
Main structure of the phosphatidylcholine, representative phosphatidylcholine groups and main fatty acid residues.

Phytosome is a relatively stable complex formed by electrostatic interaction between phospholipids, (mainly phosphatidylcholine), and plant extracts (mainly polyphenols) (Figure 2) [129]. This electrostatic effect mainly includes ion-dipole, dipole-dipole and hydrogen bonding [130]. Phospholipid complexes are more bioavailable than purified extracts because the presence of phosphatidyl cholines, a major component of cell membranes, enhances the ability of plant extracts to circulate in the body [131].

Phytosomes were developed in the late 1980s by a company called Indena, which developed a way to increase the bioavailability of drugs by complexing them to phospholipids. Many of the drug extracts currently on the market are in the form of phospholipid complexes. Phytosome technology has greatly improved the bioavailability of plant extracts, especially polyphenols. Yang et al. [132] used a simple method to prepare the rosmarinic acid-phospholipid complex (RA-PLC). The study showed that compared with natural RA, the membrane permeability and antioxidant properties of RA-PLC are significantly improved, and the biological utilization is 1.2 times higher than RA. Ravarotto et al. [133] reported that the silibinin-phospholipid complex has better anti-hepatotoxic activity than silibinin alone, and it can reduce the toxic effect of aflatoxin B1 on broilers. Similarly, there have been numerous reports that curcumin-phospholipid complexes can significantly increase bioavailability, improve pharmacokinetics, and enhance liver protection [134,135]. In the study of Marczylo et al. [136] rats given the same oral dose of free curcumin and curcumin–phospholipid complex (340 mg/kg) were found to have 5 times more drug content in plasma than the other group after 2 h. Pharmacokinetic studies in rats in another study showed that oral curcumin–phospholipid complexes had a longer half-life than oral free drugs [137]. Quercetin and polyphenol extracts from Moringa oleifera leaf have the function of improving bioavailability after complexing with phospholipids [138,139].

The application of polyphenol–phospholipid complexes in anti-cancer has gradually emerged. Narges Mahmoodi found that both silibinin and silibinin phosphatidylcholine can down-regulate the expression of HER2 on SKBR3 breast cancer cells, but silibinin–phospholipid complex had much greater inhibitory effect on cancer cells than natural silibinin (approximately 2 ~ 2.5 times) [140]. A list of polyphenols complexed with phospholipids is shown in Table 3.

### 3.2. Lipid-Based Nanoparticles

Liposome-based nanoparticles are spherical lipid particles widely used for drug delivery. The ability to encapsulate water-soluble, lipid-soluble and amphiphilic substances makes them ideal carriers for many kinds of drugs. These kinds of nanoparticles mainly include nanoliposomes and solid lipid nanoparticles [14].

#### 3.2.1. Liposomes

**Figure 3:**
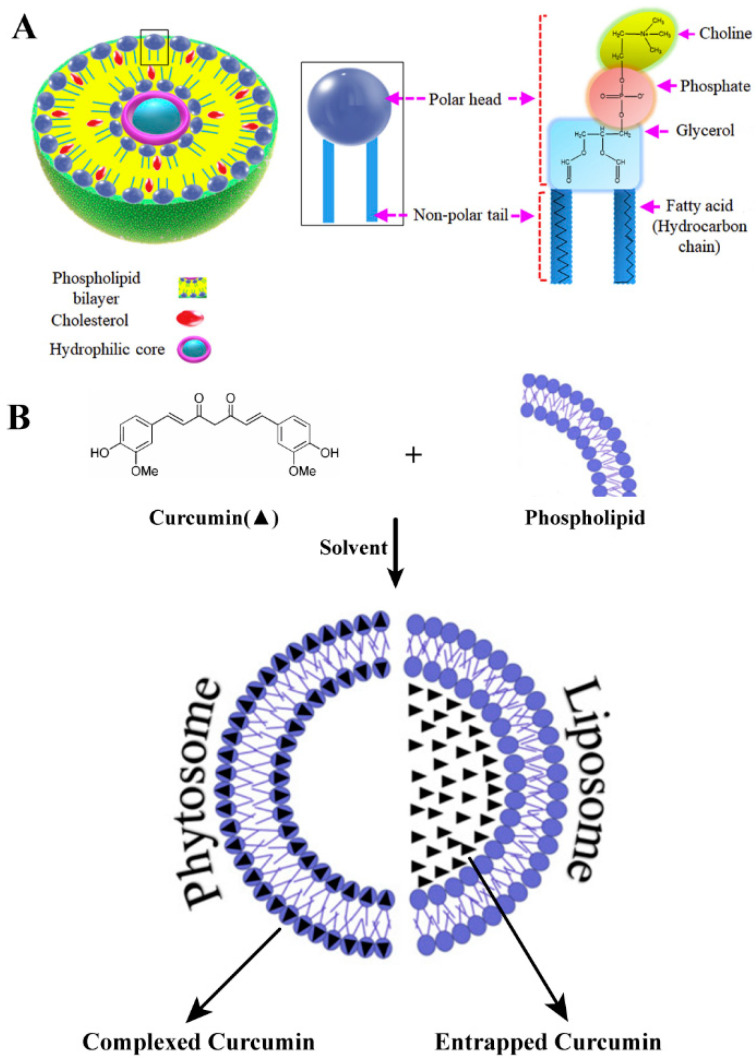
Liposome structure and drug loading diagram: (**A**) Cross-section structure of liposome, made of phospholipid and cholesterol, showing the magnified molecular structure of a phospholipid that consists of a polar head and a non-polar tail. Phospholipid head is hydrophobic and comprises choline, phosphate and glycerol, while the tail is a hydrocarbon chain that shows lipophilicity. (**B**) A schematic representation of the structure and preparation of phytosomal curcumin. Reprinted from reference [134,159]. Copyright 2016 Elsevier Masson SAS, 2019 Elsevier Ltd.

The term liposome is composed of two Greek words, lipos (fat) and soma (body or structure), meaning a membrane of fat (mainly phospholipids and cholesterol) that surrounds a water-soluble carrier or compartment [152]. Liposomes are self-assembled amphiphilic spherical vesicles with at least one phospholipid bilayer, similar to the bilayer shape on the cell membrane, which can separate the internal and external aqueous medium [14,153] (Figure 3). Liposomes vary in size, ranging from 50 nm to 1 μm, depending on composition and preparation methods. The presence of such an amphiphilic substance allows water-soluble, fat-soluble and amphiphilic compounds to be encapsulated, transferred and released [154].

There are many methods for preparing liposomes. The traditional methods include thin film hydration, reverse evaporation, injection, and heating [155]. The biggest drawback of these technologies is that the liposomes formed are large in size. The methods currently used include membrane contactor-based method, freeze drying of double emulsion method, proliposome method [156].

With the ability to improve biocompatibility, prevent drug degradation, deliver low-solubility drugs, and improve drug targeting [157,158,159], liposomes are undoubtedly a kind of promising and flexible polyphenol delivery system. In addition, it has been recognized in clinical and biological research for its role in human health, including protecting the liver, enhancing memory and reducing cholesterol intake [152]. With the deep understanding of liposomes and polyphenols, the combination of liposomes and polyphenols has attracted much attention.

Cheng reported that nano-liposomes greatly improved the bioavailability and water solubility of curcumin [160]. Vanaja et al. [161] used a thin-film method to load resveratrol into liposomes. Compared with free resveratrol, resveratrol packed in liposomes was more active in cells in vivo and had more significant antioxidant effect. A novel remote loading approach using chemically modified β-cyclodextrin was applied to incorporate curcumin into liposomes [162]. The results proved that the encapsulation of the nanoparticles significantly improved the bioavailability of curcumin, and the complexation of cyclodextrin further increased the encapsulation efficiency of curcumin.

Huang and coworkers co-loaded curcumin and resveratrol in liposomes [163]. Infrared spectroscopy and fluorescence techniques demonstrated that curcumin was connected to the hydrophobic acyl chain region of liposomes, while resveratrol was located in the polar hydrophilic region. The study also investigated the physical and chemical properties of the liposomes. The results showed that, when the ratio of curcumin to resveratrol was 5:1, the encapsulation rate and the antioxidant activity was the highest. Similarly, co-loading quercetin and resveratrol, using liposome as the nanocarrier, enhanced the cellular uptake and ROS scavenging ability than single drug loading [164].

However, the limitation of liposome is that it has a short half-life and can be easily oxidized and hydrolyzed. It was previously reported that the intravenous administration of resveratrol resulted in a short t_1/2_, ranging from 7.8 to 33 min [165]. Therefore, many modified liposomes came into being. In order to increase the half-life and extend the blood circulation time, Caddeo et al. [166] grafted polyethylene glycol chains (PEGylated liposomes) at the ends of liposomes to deliver resveratrol. Drug release results indicated that the half-life of polyethylene glycol modified liposomes was increased by about nine times, while the inherent antioxidant activity was still maintained. By mixing with various biopolymers, the storage stability of liposomes was improved [167]. The biopolymers include anionic (such as arabinose and whey protein) and cationic (such as chitosan). It was also proven that the encapsulation rate of polyphenols in liposomes and their antioxidant activity will increase with the addition of biopolymers.

#### 3.2.2. Solid Lipid Nanoparticles (SLNs)

Solid lipid nanoparticle is a solid colloidal drug delivery system which is made by wrapping or inserting drugs in lipid nucleus with natural or synthetic lipids, such as lecithin and triglyceride. The emergence and development of SLNs successfully make up for the limitations of traditional nanocarriers (such as nanoemulsion and liposomes), with advantages including controlling drug release and targeting, increasing drug stability, high drug loading, low toxicity, and loading of hydrophilic lipophilic drugs.

SLNs coated with resveratrol were prepared by Pandita and coworkers using solvent diffusion - solvent evaporation method [168]. The encapsulation rate of resveratrol in nanoparticles was 88.9±3.1%, and the release time in vitro could be extended to 120 h. It was also found that the use of solid lipid formulations increased the bioavailability of oral resveratrol by eight times compared to drug suspensions. Similarly, glyceryl behenate-based SLNs were used for the encapsulation of resveratrol to explore its brain targeting ability [169]. Cytotoxicity experiments demonstrated that SLNs had the same antitumor effect as free resveratrol, while the drug biodistribution in mice indicated that SLN greatly increased the concentration of resveratrol in brain cells.

Curcumin was also incorporated within chitosan coated SLNs by homogenization and ultra-sonication technique [170]. It showed that the oral bioavailability of nanoparticles was more improved than that of curcumin suspension, and the reason was not only that the encapsulation prevented the drug from being degraded by enzymes, but also that the chitosan could be easily absorbed. The production of such formulations could further expand the use of curcumin in food and nutraceuticals.

SLN was also used to improve the bioavailability of quercetin by carrying transferrin that enabled it to be delivered to the brain at designated points to study the role in Alzheimer’s disease [171]. It was found that the nanoparticles could inhibit the formation of fibrils and reduce the amyloid accumulation of peptides.

### 3.3. Niosomes

**Figure 4:**
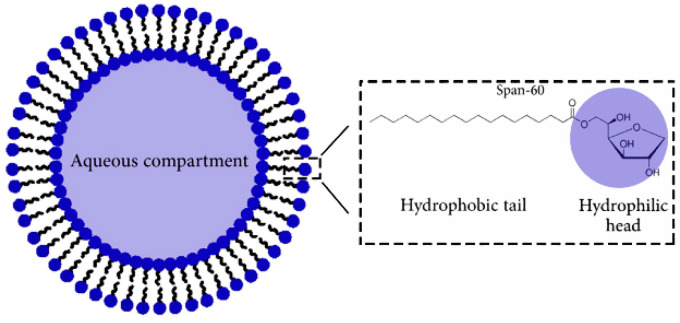
Schematic representation of niosome prepared by sorbitan monostearate (Span-60). Redrawn from reference [176]. Copyright 2014 Elsevier B.V.

Liposomes have the disadvantages of high production cost, poor chemical stability, and impure phospholipid content. With similar structures to liposomes, niosomes can perfectly avoid those disadvantages and become promising polyphenol delivery systems that have been used for continuous, controllable and targeted drug delivery of polyphenols and other drugs [172,173,174]. Most of the niosomes are composed of non-toxic non-ionic surfactants (mainly alkylamides, alkyl esters, and fatty acid esters), and contain cholesterol or its derivatives and charged molecules. The presence of cholesterol increases the rigidity of nanoparticles, while the presence of charged molecules contributes to the stability during the preparation (Figure 4) [174,175,176].

The preparation methods of niosomes include the ethanol injection method, transmembrane pH gradient method, reverse phase evaporation, microfluidization, lipid layer hydration, etc. Due to the unique structure, niosomes can be used to load and deliver hydrophilic and hydrophobic substances. Lu et al. designed Span60-Rh40-based niosomes for loading flavonoids to improve the solubility, stability and penetration [177]. It was demonstrated that niosomes significantly improved the solubility and light stability of quercetin. The skin water-locking effect of quercetin-niosomes was almost three times higher than that of free quercetin solution. Another group extracted polyphenol-rich propolis with ethanol and prepared propolis-loaded niosomes (PLNs) with different concentrations of Span 60 and cholesterol to enhance the local antibacterial effect of propolis. The use of niosomes significantly enhanced the antibacterial activity of propolis against *Staphylococcus aureus* and *Candida albicans*. The enhancement of antibacterial activity attributes to the fact that niosomes can directly interact with the bacterial envelope, thus facilitating the entry of antibacterial components in propolis into cells [178]. To increase chemotherapeutic efficacy while reducing toxic effects, a rational design for synergy-based drug regimens is essential. Alemi et al. found that the combination therapy of paclitaxel with curcumin using PEGylated noisome delivery enhanced cytotoxicity against MCF-7 cells [179].

### 3.4. Protein-Based Nanoformulations

Protein-based drug delivery system has high nutritional value and good functional characteristics, making it continue to develop in the food industry.

#### 3.4.1. Casein-Based Nanoparticles

Casein is the predominant protein in milk (approximately 80%), composed of αs1-, αs2-, β-, and κ-caseins, with unique hydrophilic and hydrophobic domains. These proteins self-assemble in the presence of calcium phosphate to form a spherical colloid with a diameter of 50–500 nm (average diameter 150 nm). Because the casein structure is rich in proline and can adapt to changes in its environment, it is defined as a rheological protein [180,181].

Casein has many properties that favor its use in drug delivery systems, including exceptional surface activity, excellent emulsification and self-assembly properties, and excellent water binding ability [182]. In addition, casein can interact with many molecules to form stable complexes and conjugates. These unique properties make it an excellent choice for drug delivery systems.

Studies have shown that β-casein encapsulation increases the solubility of curcumin by at least 2500-fold, and the presence of this casein micellar protein enhances the cytotoxicity of the drug to human leukemia cells K-562. Furthermore, curcumin–casein showed significantly higher antioxidant activity than free curcumin [183]. Luo et al. [184] prepared rutin-sodium caseinate/pectin complex nanoparticles by acidification and heat treatment. It was found that heating not only improved the rate of nanoparticles formation, but also significantly improved the rate of rutin encapsulation. The presence of pectin delayed the hydrolysis of sodium caseinate by pepsin and allowed the controlled release of rutin in the gastrointestinal tract. In another study, rutin was coated with sodium caseinate and trehalose complex, and an analysis revealed that rutin was amorphous after addition of trehalose and pH adjustment. The powder produced by co-precipitation with sodium caseinate contained large amounts of rutin [185]. Ghayour et al. [186] encapsulated curcumin and quercetin using the coating method. The encapsulation efficiency of both compounds was greater than 90%, and their solubilities in nanoparticles were higher than those of the free polyphenol molecules. In addition, polyphenol-casein nanoparticles were more cytotoxic towards MCF-7 human breast cancer cells than unloaded molecules.

#### 3.4.2. Gelatin Nanoparticles

**Figure 5:**
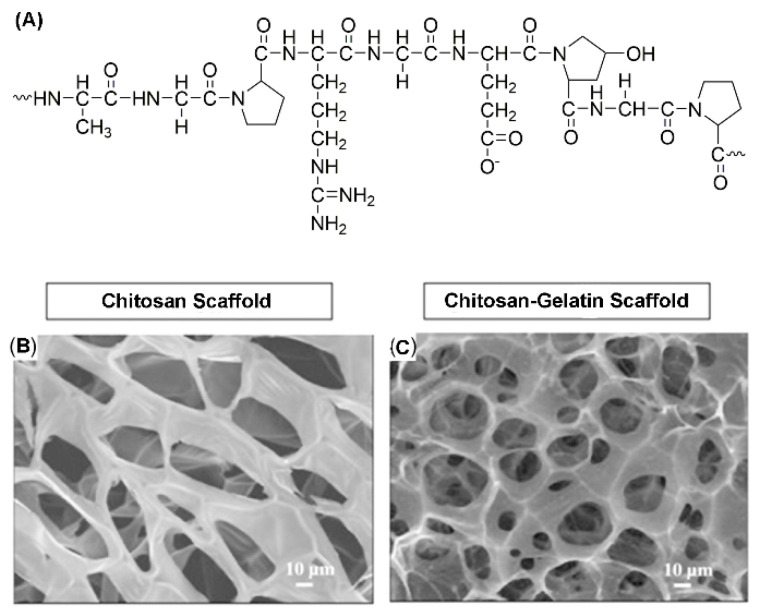
(**A**) The chemical structure of the gelatin. (**B**) Scanning electron microscopy (SEM) images of pure chitosan scaffold and (**C**) chitosan–gelatin scaffold. Redrawn from reference [195]. Copyright 2009 Elsevier Ltd.

Gelatin is a water-soluble protein, that is prepared from collagen by acid or alkali hydrolysis (Figure 5) [187]. The US Food and Drug Administration generally believes that it is safe for use in medicine, cosmetics and food [188]. If sulfuric acid or hydrochloric acid is used to hydrolyze egg collagen, the gelatin obtained is of type A, with an isoelectric point of about 9. Correspondingly, if an alkaline solution is used to hydrolyze collagen, the gelatin produced is of type B with an isoelectric point of about 5 [189]. Gelatin is a polyamphoteric electrolyte with hydrophobic groups. Apart from being cheap and easily obtained, gelatins have good biocompatibility and biodegradability. First, because it is a deformable protein, the antigenicity of gelatin is relatively low compared with that of collagen. Second, gelatin produces no harmful by-products after enzymatic hydrolysis. In addition, the intrinsic protein structure of gelatin and the presence of many functional groups enable it to be coupled with many crosslinking agents and ligands. This has far-reaching significance for the development of targeting vectors [190,191]. Methods of preparing gelatin nanoparticles include desolvation, coacervation-phase separation, emulsification-solvent evaporation and nanoprecipitation [192,193,194].

Gelatin nanoparticles have been used for the effective delivery of a variety of drugs, including polyphenols. Shutava et al. [196] encapsulated several polyphenolic compounds—epigallocatechin gallate (EGCG), curcumin, tannic acid and catechin—in gelatin nanoparticles, and modified the nanoparticle surface with a layer-by-layer polyelectrolyte shell to increase the stability of the gel and control the release of polyphenols. The release of EGCG from the gel was found to be up to 8 h, much longer than the minutes when in the free state. Furthermore, nanoparticle-coated EGCG retained its biological activity in MB-MD-231 breast cancer cells. Karthikeyan et al. [197] prepared resveratrol-gelatin nanoparticles by agglomeration. We understand that the nanoparticles may induce the apoptosis of cancer cells by affecting the expression of p53, p21, caspase-3, Bax, Bcl-2 and NF-κB. To investigate the release of free tea polyphenols and nanoparticulate tea polyphenols in fatty foods from gelatin films, chitosan nanoparticles were prepared using the ionic gel method and bonded to gelatin films. It was found that the amount of tea polyphenols released from the gum film was related to the type of fatty food used and the encapsulation rate of the tea polyphenols. The presence of chitosan hydrochloride increased the diffusion time of tea polyphenols in the simulant, and the association was positively correlated [198].

#### 3.4.3. Whey Protein (Mainly β-lactoglobulin) Nanoparticles

Whey protein is extracted from whey, a by-product of cheese production, and is composed of many proteins, including α-lactalbumin (α-la), β-lactoglobulin (β-lg), bovine serum albumin (BSA) and immunoglobulins, and lactoferrin [199]. Whey protein is considered to be ideal for encapsulating and delivering compounds such as polyphenols, due to its high safety, low cost, high nutritional value, and diverse functions [200]. Among them, β-lactoglobulin is the most widely used. Beta-lactoglobulin is the main whey protein and gelling agent in milk. It is present in most mammalian milk, but not in human milk, and is a small globular protein, consisting of only 162 amino acids, with a molecular weight of 18.3 kDa. Whey proteins have several transfer-friendly functions, such as binding to hydrophobic active substances, gelation and emulsification. In addition, whey protein is resistant to pepsin, so it is beneficial for the oral transport of polyphenols and other substances [201].

Shpigelman et al. [202] delivered EGCG with thermally modified lactoglobulin, and found that the correlation constant of EGCG with preheated protein was about 3.5 times higher than that of the natural protein. Because the size of EGCG-lactoglobulin nanoparticles is relatively small, they can maintain good transparency for the processing and preparation of transparent drinks. In addition, lactoglobulin encapsulation greatly protected the antioxidant activity of EGCG, and the degradation of EGCG in nanoparticles was 3.2 times slower than that of free EGCG within eight days. Li et al. [203] delivered curcumin with β-lactoglobulin and nanoemulsion as the carrier. The results showed that the water solubility, pH stability and permeability of curcumin were significantly improved by binding to β-lactoglobulin. The curcumin-β-lactoglobulin complex was resistant to pepsin, but sensitive to trypsin. In another study, whey protein-safflower complex was prepared under different pH conditions. The emulsification, thermal stability and oxidation resistance of the complex were improved compared with whey protein alone [204].

A large number of studies have shown that the complexation of polyphenolic compounds with whey protein can not only improve the bioavailability of polyphenolic compounds, but also improve the functional properties of whey protein. Chen et al. [205] studied the effect of lotus heart proanthocyanidin (LSPC) on the stability of carotene whey protein nanoemulsion. The results proved that the addition of LSPC significantly improved the chemical stability of the nanoemulsion, and the use of whey protein enhanced the antioxidant activity of LSPC. Morais has reported on the interactions of whey protein with (−)-EGCG and caffeic acid (CA) at different pH values and the antioxidant capacities of the complexes [206]. The study showed that the complexation of the two polyphenols with whey protein altered the protein structure under both acidic and neutral conditions, and the effect of CA was more significant. The complexation of whey protein with CA had a synergistic effect on its reducing power and antioxidant capacity. In contrast, the complexation of whey protein with EGCG inhibited its reducing power and antioxidant capacity. As previously mentioned, whey protein complexation prevents CA and EGCG from being degraded during digestion, increasing the oral availability of the phenolic compounds.

### 3.5. Polymeric Nanoparticles

Polymer nanoparticles are spherical or irregularly shaped colloidal particles formed by polymer materials [207]. They have high stability, uniform particle size, high drug loading rate, high biocompatibility, good drug release control and are easily produced in a factory. These advantages make them widely used for the encapsulation of natural extracts, including polyphenols [208,209,210]. Polymer systems are mainly divided into two categories: natural polymers (proteins and polysaccharides) and synthetic polymers.

#### 3.5.1. PLA/PLGA

**Figure 6:**
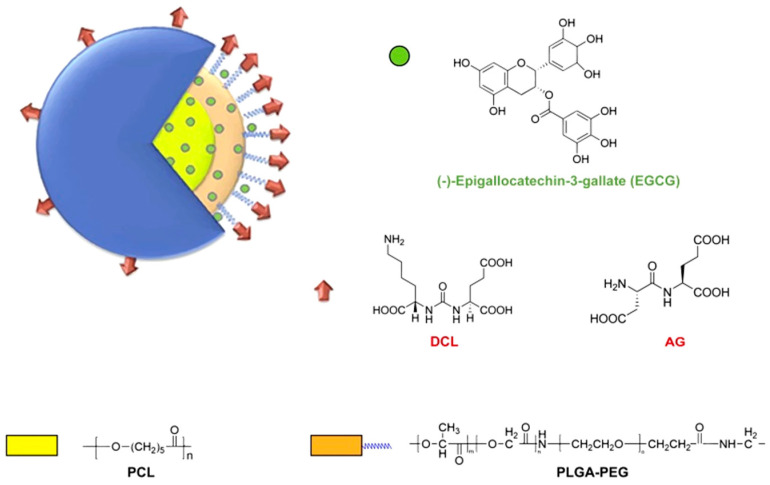
Schematic representation of the designed targeted EGCG NPs. Chemical structure of (-)- epigallocatechin-3-gallate (EGCG), the PEGylated PLGA polymers (PLGA-PEG), and the targeting ligands DCL and AG. Adapted from reference [213].

Polylactic acid (PLA) and polylactic acid-glycolic acid copolymer (PLGA) (Figure 6) have been widely used in drug delivery systems, due to their excellent biocompatibility, biodegradability and particle size control [211,212].

The use of PLA/PLGA nanoparticles improves the water solubility and poor stability of polyphenols. The release kinetics of polyphenol-PLA/PLGA nanoparticles in vitro show rapid release are at first followed by slow release, which demonstrates the controlled release of polyphenol compounds from PLA/PLGA nanoparticles [214]. Similar to other nanoparticles, PLA/PLGA also enhances the functional activity of polyphenols, such as anti-inflammatory, antioxidant and anti-cancer properties [215,216,217].

Although PLA has many advantages, it has certain limitations as a drug delivery system due to its hydrophobicity and low chemical stability. These limitations are particularly obvious when used for oral administration, because the nanopariticles are very likely to be intercepted and quickly cleared by mucus and cilia [218]. To avoid these limitations, PLA/PLGA nanoparticles are often modified by other polymers (such as polyethylene glycol (PEG) or chitosan) or are used in combination with other polymers. Studies have shown that a PEG coating on the nanoparticle surface can ensure rapid passage through the mucus layer, and significantly improve hydrophilicity and stability [219]. The toxicity of resveratrol-loaded PEG-PLA polymer nanoparticles to CT26 colon cancer cells was the same as that of free resveratrol, but encapsulation significantly increased its stability and cycling time, conferring significant anti-tumor effects [220]. Mixed micelles prepared from mPEG-PLA/TPGS enhanced the absorption of curcumin from the gastrointestinal tract, and its oral availability was much higher compared to curcumin suspension [221]. New targeted nanoparticles coated with EGCG exhibited excellent anti-proliferative activity in vitro, and tumor inhibition by the nanoparticles was significantly enhanced compared with the natural compounds [222]. In addition to PEG modification of PLGA, other polymers such as chitosan are often used as modifiers to increase the stability of nanoparticles. Chitosan oleic acid was applied to PLGA containing curcumin by emulsification and solvent evaporation, resulting in PLGA nanoparticles that were more stable than polymer micellar ones [223].

#### 3.5.2. Chitosan

Chitosan is a semi-synthetic polysaccharide obtained by the deacetylation of chitin, which is widely present in nature, and is the only alkaline polysaccharide. Chitosan has received increasing attention, due to its good biocompatibility, degradability, non-antigenicity, high permeability, non-toxicity and good film-forming properties [224,225]. In addition, it has adhesive properties, and can reversibly open the tight junctions between epithelial cells, thereby promoting paracellular transport between cells [226]. Moreover, chitosan readily interacts with negatively charged polymers, conferring targeting properties to nanoparticles.

Chitosan has been widely used as a carrier in drug delivery systems. By increasing the release time and adhesion of polyphenols, chitosan nanoparticles significantly improved the functional activity and oral availability of the active substances. Encapsulation by chitosan enables the available concentration of green tea polyphenols to meet physiological requirements during treatment. The EGCG nanoformulation has an 8-fold dosage advantage compared with natural polyphenols. In addition, EGCG nanoparticles significantly induced apoptosis in human melanoma cells [227]. Similarly, the nanoparticles inhibited the growth of gastric cancer cells and reduced the expression of vascular endothelial growth factor protein by controlling the release of EGCG in gastric acid [228]. Studies have shown that the stability of chitosan-gum arabic polysaccharide nanoparticles loaded with curcumin in a simulated gastrointestinal environment is significantly improved. The release of curcumin is delayed, and the antioxidant activity of the active substance is also significantly enhanced [229]. Compared with free curcumin, curcumin-chitosan nanoparticles are more easily absorbed by colon cancer cells. The adhesion also prolongs contact time, resulting in a decrease in cell viability [230].

Although chitosan has many excellent features, properties required for some specific applications can be obtained by modifying it. Curcumin diethyl ester encapsulated with chitosan-alginate nanoparticles can be stored stably at 4 °C for three months, and the nanoparticles significantly improve cellular uptake by Caco-2 cells [231]. Studies have shown that folate-modified chitosan nanoparticles are able to target breast cancer cells, which complements curcumin-induced apoptosis of breast cancer cells. In addition, folic acid-modified chitosan nanoparticles are also responsive to pH changes. Compared with natural resveratrol, resveratrol loaded in carboxymethyl chitosan exhibits prolonged absorption and duration of action. Its antioxidant activity and bioavailability are also significantly increased [232].

In addition to the simple encapsulation of polyphenols, chitosan is increasingly popular in the food packaging industry, due to its film-forming ability and biodegradability [233]. It has been reported that pure chitosan has good mechanical strength and air permeability, but its antioxidant performance is not ideal. Addition of polyphenolic compounds with antioxidant properties can solve such problems [234]. A large number of studies have shown that the addition of polyphenols greatly improves resistance to oxidation, thermal stability, mechanical strength and pH-responsiveness of chitosan membranes [235,236,237].

#### 3.5.3. Cyclodextrins

**Figure 7:**
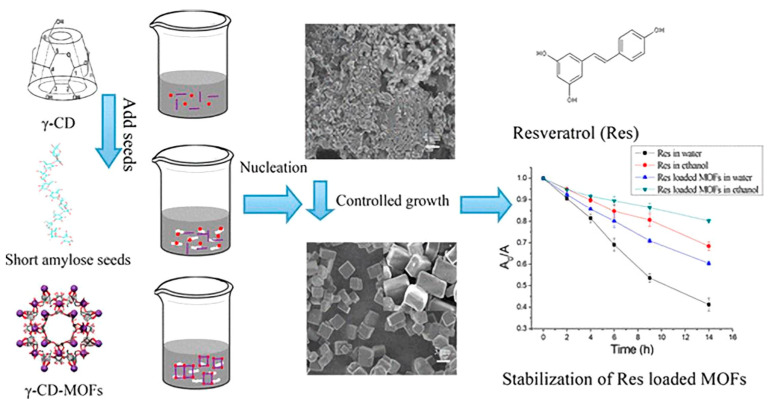
Schematic representation of the size-controlled synthesis of γ-CD-MOFs through facile and green seed-mediated method. Adapted from reference [244]. Copyright 2018 American Chemical Society.

Cyclodextrin (CD) is the general term for a series of circular oligosaccharides produced by amylose under the action of cyclodextrin glucosyl transferase. As a natural carrier, it has been widely used in the pharmaceutical and food industries for encapsulation of bioactive compounds to improve their bioavailability and stability. Cyclodextrins are circular oligosaccharides with hollow pyramidal structures connected by 6, 7 or 8 glucose residues via 1,4 glycosidic bonds. They are produced by enzymatic hydrolysis of starch [238]. In nature, they exist as α-, β- and γ-CDs (Figure 7). The hydrophobic inner cavities of CDs can be used to encapsulate phenolic compounds to protect them from the external environment, such as pH, light, temperature and oxygen.

Studies have confirmed that the encapsulation of resveratrol into a cyclodextrin-based metal-organic framework greatly improves its stability [239]. It is controversial whether the complexation of polyphenols by cyclodextrin will affect their biological activity. It has been found that catechins and cyclodextrins interact through intermolecular O–H...O hydrogen bonds. The establishment of this host-guest relationship helps to improve the antioxidant capacity of polyphenols [240]. Similarly, when β-cyclodextrin is used to extract and protect phenolic compounds in olive oil, it does not affect their in vitro bioavailability [241]. In contrast, it was found that the oxygen radical absorbance capacity-fluorescein (ORAC-FL) value of the (+) catechin-cyclodextrin complex was lower than that of free catechin, indicating that complexation reduced the antioxidant activity of polyphenols [242]. This difference may be due to a variety of factors, such as the method, temperature, and matrix used in the complexation. Ho et al. found that catechin complexes were more stable in a solid matrix compared to semi-solid or liquid matrixes. The food matrix will affect the stability and recovery rate of catechins from the inclusion compound [243].

#### 3.5.4. Hydrogels

A hydrogel is a three-dimensional, porous, shape-retaining, chemically or physically cross-linked highly water-soluble polymer [12]. The characteristics of hydrogels include mechanical resistance, swelling potential and moisture retention capacity [245,246].

**Figure 8:**
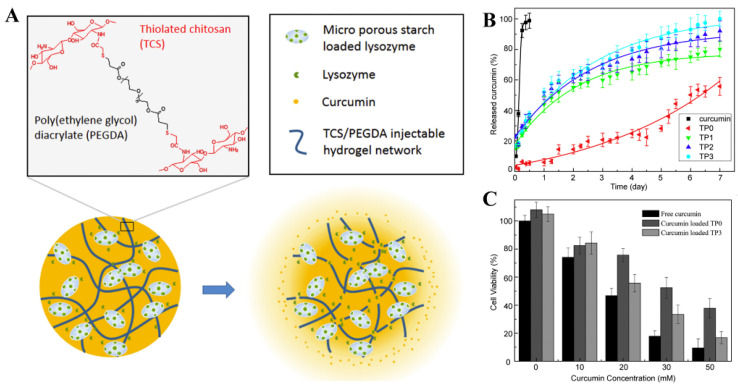
(**A**) Scheme of thiolated chitosan/poly(ethylene glycol) diacrylate (TCS/PEGDA) injectable hydrogel for localized intratumoral delivery of anti-cancer drugs. (**B**) Curcumin release behavior from TCS/PEGDA injectable hydrogel in PBS buffer at 37 °C with shaking (100 rpm). TP0 is the gel with micro porous starch but without lysozyme; TP1, TP2 and TP3 are the gels with micro porous starch adsorbed 0.46, 0.60 and 0.75 g/g lysozyme, respectively. (**C**) HepG2 cells viability determined using MTT assay when incubated with free curcumin, and curcumin loaded TCS/PEGDA injectable hydrogels with (TP3) or without (TP0) lysozyme, respectively. Adapted from reference [252]. Copyright 2017 Elsevier B.V.

When anti-inflammatory polyphenols are added to hydrogels that mimic the natural extracellular matrix and hydration environment, they form an ideal material for skin wound healing. Resveratrol-polypeptide-hydrogel nanoparticles are not cytotoxic and inhibit the macrophage release of pro-inflammatory factors to accelerate wound healing [247]. Animal experiments and molecular mechanism studies have shown that PVA/alginate hydrogel particles coated with tea polyphenols can promote wound healing in diabetic rats by regulating the PI3K/AKT signaling pathway [248]. Similarly, a newly developed hydrogel based on plant polyphenols, tannins and polypyrrole chains can stimulate tissue repair after bone marrow injury [249]. Of course, other biological activities, such as the antibacterial effects of polyphenols, are also fully compatible with hydrogel encapsulation. One study showed that PEG-lysozyme (LZM) polyphenolic hydrogels were more flexible than the original PEG-LZM, and the addition of polyphenols significantly improved the antibacterial and anti-inflammatory properties of the hydrogels [250]. The edible film prepared from tea polyphenols and calcium alginate gel has anti-oxidant and anti-inflammatory functions. The ductility, fracture strain, and air permeability of the film increases as the polyphenol content is increased [251]. Another study confirmed that injectable hydrogels based on curcumin, thiochitosan and polyethylene glycol diacrylate can promote the apoptosis of cancer cells and effectively delay tumor growth (Figure 8) [252].

#### 3.5.5. Dendrimers

A dendrimer is a monodisperse, three-dimensional, hyper-branched radial symmetric polymer with host-guest capabilities [253]. Its core structure is a cavity that can contains biologically active components, and the branches can be modified or complexed with other compounds [254]. In addition, dendrimers can simply pass through biological barriers. Among the known dendrimers, PAMAM is the most widely used in drug delivery systems. Using silicon-PAMAM hybrid nanoparticles to load procyanidins, proanthocyanidins were completely released after six days, and the cytotoxicity reached at 87.9% after 134 h. Moreover, the mixed nanoparticles were found to have no toxicity towards normal cells [255]. Studies have shown that resveratrol, genistein and curcumin combine with PAMAM-G3 and PAMAM-G4 through hydrophilic, hydrophobic and hydrogen bonds to form stable complexes. The larger the nanoparticles, the higher the loading and stability of polyphenols, increasing their bioavailability [256,257].

In addition to PAMAM, other dendrimers have also been used to protect and deliver polyphenols, thus improving their bioavailability and stability. Gallic acid (GA) was loaded into the fifth-generation polyester dendrimer, and the large surface area of the nanoparticles significantly improved GA retention time and biological efficacy. Moreover, the antioxidant capacity of encapsulated gallic acid was four times higher than that of free GA [258]. The binding of dendritic plant glycogen (PG) significantly increased the solubility, in vitro permeability (Caco-2 monolayer permeability) and anticancer effects (HeLa cells) of curcumin compared with natural curcumin [259].

### 3.6. Micelles

Polymer micelles composed of amphiphilic polymer molecules are a new type of polyphenol carrier. Their hydrophobic core can be used to encapsulate water-insoluble substances, and the hydrophilic corona that protects the core can escape removal by the reticuloendothelial system (RES), increase the circulation time, and avoid interaction with blood components [260,261,262]. The micelles have diameters of 20–100 nm, which enables their movement from the tumor blood vessel wall into cancer cells [263]. Micelles are often used to encapsulate doxorubicin and polyphenolic compounds to increase the therapeutic effect while protecting the heart [264]. A study loaded resveratrol and quercetin Pluronic^®^ F127 micelles (mRQ) with doxorubicin hydrochloride and found that the heart was protected by the combination with the two polyphenolic compounds. In addition, the presence of mRQ did not affect the caspase activity of human ovarian cancer cells (SKOV-3), but significantly reduced the caspase activity of rat cardiomyocytes (H9C2) [265]. Replacing the quercetin in the nanoparticles with curcumin gave similar results. The combined use of polyphenols and doxorubicin can reduce cardiac toxicity by reducing apoptosis and ROS production, and increase the efficacy of doxorubicin against ovarian cancer cells [266]. A large number of research results have shown that many biological activities of polyphenols are fully reflected by encapsulation in micelles. Curcumin encapsulated in monomethoxy-polyethylene glycol-chitosan-S-S-hexadecyl micelles effectively promoted the accumulation of active substances in cells and significantly down-regulated the expression of tumor necrosis factor. In addition, the nanoparticles also showed good anti-inflammatory effects in the tumor microenvironment [267]. Compared with free doxorubicin, doxorubicin/curcumin colloidal nanoparticles in human liver cancer SMMC 7721 cells exhibit prolonged release, a higher rate of cell apoptosis, and a stronger anti-angiogenesis effect [268].

**Figure 9:**
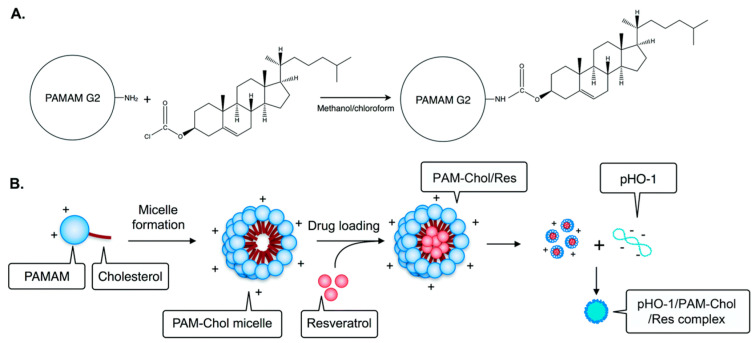
Synthesis of cholesterol-conjugated PAMAM (PAM-Chol) (**A**) and preparation of the pHO-1/PAM-Chol/Res complex (**B**). The heme oxygenase-1 gene (pHO-1). Adapted from reference [269]. Copyright 2018 Royal Society of Chemistry.

In another study, resveratrol was loaded into cholesterol-polyamide micelles by solvent evaporation. The micelles reduced the production of pro-inflammatory factors in the lungs by the inhibiting nuclear transposition of NF-κB, demonstrating the anti-inflammatory effect of resveratrol (Figure 9) [269]. Washington et al. [270] used poly(ethylene glycol)-b-poly(ε-caprolactone) (PEG-b-PCL) and poly(ethylene glycol)-b-poly(γ-benzyl-ε-caprolactone) (PEG-b-PBCL) amphiphilic copolymer micelles loaded with doxorubicin and resveratrol. The encapsulation efficiency of doxorubicin in PEG-b-PBCL micelles was only 31%, while co-loading increased the encapsulation rate to 87.7%. In addition, the composite drug-loaded micelles were more cytotoxic to HeLa cells than micelles containing only DOX.

### 3.7. Nanoemulsion

Nanoemulsion refers to a system composed of two immiscible liquids, that is divided into an internal phase (or dispersed phase) and an external phase (or continuous phase). The internal phase is dispersed in the external phase [271]. Emulsions can be divided into micro- (10–100 nm), mini (nano)- (100–1000nm), and macro-emulsions (0.5–100mm) according to size [272], and many properties of the emulsion, such as stability, color and stability, are closely related to droplet size. Nanoemulsion is located between normal emulsion and microemulsionQ, and the average diameter is mostly less than 100 nm.

Nanoemulsions have been used to encapsulate a variety of polyphenolic compounds, such as curcumin, due to their small size, large surface area, high optical clarity, good stability, and ability to improve drug bioavailability. Yu et al. [273] prepared a nanoemulsion with a curcumin organogel. Pharmacokinetic analysis in mice showed that the oral availability of curcumin from the nanoemulsion was nine times higher than that of unformulated curcumin, and the digestion of the nanoemulsion in the gastrointestinal tract was significantly faster than that of the organogel. Another team used ultrasound to prepare a curcumin-nanoemulsion. In a gastrointestinal simulation experiment, the release rate of curcumin from the nanoemulsion was slower, because the nanoemulsion was not easily hydrolyzed by pepsin, and pancreatin can cause its release [274]. Similarly, Zou et al. [275] studied the potential of three nano-drug delivery systems, nanoemulsion, zein nanosuspension and nanoliposomes, to improve curcumin bioavailability. The study found that curcumin loaded into nanoemulsion was most effective for gastrointestinal absorption. In one study, EGCG encapsulated in an oil/water nanoemulsion exhibited significantly increased anticancer activity in vitro compared with free EGCG, and the droplet size of the nanoemulsion hardly changed over 14 days [276]. In another study, the authors investigated the effects of nanoemulsion encapsulation on the physical and chemical properties, biological activity, and epithelial permeability of EGCG. Compared with unencapsulated catechins, the bioaccessibility of EGCG-nanoemulsion increased by 2.78 times. In addition, the intestinal permeability of EGCG was significantly increased. These results showed that a soybean protein nanoemulsion could improve the stability, bioaccessibility and permeability of green tea catechin [277].

Although nanoemulsions have significant advantages, they are unstable at low pH, and their small size and liquid nature make drug release difficult to control. In addition, the preparation of nanoemulsions requires specialized equipment, so there is a perception in the food industry that nanoemulsions are unprofitable [14].

### 3.8. Metal Nanoparticles

Compared with organic nanoparticles, inorganic nanoparticles have unique characteristics, such as good controllability of size and shape, large specific surface area, and imaging potential. They also enable targeted drug delivery and synergistic therapy. These characteristics make them well suited for drug delivery. Among all inorganic nanoparticles, metal nanoparticles are the most widely used. Metal nanoparticles are composed of pure metals such as gold, silver or platinum, with a size of 1–100 nm. In recent years, they have attracted increasing attention due to their huge potential in drug delivery systems [278,279].

#### 3.8.1. Gold Nanoparticles (AuNPs)

**Figure 10:**
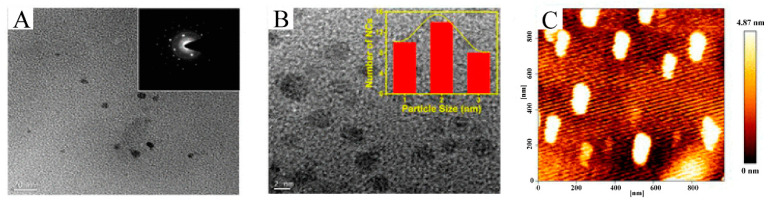
Morphology characterization of CUR-AuNCs: (**A**) TEM of CUR-AuNCs; the inset image is SAED pattern. (**B**) HR-TEM; arrow indicates the collections of atoms to form a cluster. (**C**) Bio-AFM height image of CUR-AuNCs. Adapted from reference [284]. Copyright 2018 American Chemical Society.

Gold nanoparticles are crystal structures composed of metal gold atoms, and are the most widely used metal nanoparticles, with many properties that make them the most promising nanomaterials in biomedical fields such as biosensors, molecular imaging and drug carriers. They are 1–100 nm in size, and form a variety of shapes such as ball, bar and cage. In addition, they are non-toxic, biocompatible, are negatively charged and easily functionalized by other biomolecules [280,281,282]. Traditional gold nanoparticles are synthesized using chemicals that are harmful to human health and the environment [283]. In recent years, numerous gold nanoparticles have been prepared using plant active compounds that are friendly to the human body and the environment, such as polyphenols [284]. Curcumin (Cur) is the most widely used plant active compound in the preparation of gold nanoparticles (Figure 10).

Compared with free curcumin, Cur-AuNPs showed significantly increased cytotoxicity to colon and breast cancer cells. The conjugated nanoparticles showed no toxicity to normal kidney cells, exhibiting excellent biocompatibility [285,286]. Compared with other nanoparticles, gold nanoparticles are composed of a small number of atoms, and fluoresce under visible light (550 nm) irradiation. This feature makes them useful for biological imaging. Compared with the control group, curcumin-conjugated gold clusters (Cur-AuNCs) significantly inhibited the migration of HeLa cells and exhibited significant cytotoxicity [284]. Other polyphenol compounds can also participate in the synthesis of gold nanoparticles. Compared with free EGCG and citrate-gold nanoparticles, AuNPs prepared with EGCG as a reducing agent more potently inhibit the growth of cancer cells such as PC3 and MDA-MB-231 and induce apoptosis. Resveratrol-gold nanoparticles have good stability, photothermal performance and antioxidant capacity. Moreover, under laser irradiation, the nanoparticles block the cancer cell cycle and inhibit cell division, leading to apoptosis [287]. Studies have also evaluated the effects of quercetin-gold nanoparticles in breast cancer cell lines. It was found that the nanoparticles inhibited the angiogenesis and metastasis of breast cancer cells by targeting the EGFR/VEGFR-2 signaling pathway. Compared with free quercetin, nanoparticles induce more apoptosis of cancer cells [288,289].

#### 3.8.2. Silver NPs (Ag NPs)

Similar to gold nanoparticles, silver nanoparticles also have anti-inflammatory, antibacterial, anti-cancer and other biological activities [290]. In recent years, researchers have been interested in the use of plant extracts, such as polyphenols, as reducing agents and stabilizers for green synthesis of silver nanoparticles [291]. Green synthetic methods embody the advantages of cost-effectiveness, eco-friendliness and biocompatibility [292].

The antimicrobial activity of AgNPs has attracted much attention. AgNPs synthesized using hydrolyzate rich in polyphenols have good activity against bacteria and fungi [293]. Silver nanoparticles synthesized from the aqueous extract of laurel stem had significant activity against both Gram-positive and Gram-negative bacteria [294].

Other biological properties of AgNPs synthesized from polyphenolic plant extracts, such as anti-inflammatory, antioxidant and anticancer activities, have also attracted increasing attention. Polyphenols (extracted from Cornus officinalis)-silver nanoparticles inhibit the production of pro-inflammatory cytokines by inhibiting the activation of NF-κB in macrophages, thus showing good anti-inflammatory activity in the treatment of psoriasis [295]. AgNPs prepared using the water extract of Lintong leaf is a good analgesic and muscle relaxant, and can be used for pain care [296]. Although silver nanoparticles themselves have antibacterial and anticancer activities, the role of polyphenols and other plant extracts in AgNPs should not be ignored. Studies have shown that these active substances can bind to the final AgNPs. In fact, polyphenols and silver have a synergistic relationship in respect of antioxidant, antibacterial, anti-inflammatory and other properties [297,298].

## 4. Conclusions and Future Trends

As some of the most widely distributed plant active compounds, phenolic compounds have many functions that are beneficial to human health. However, the low solubility, poor stability and low bioavailability of these compounds greatly limit their applications in food and medicine. Encapsulation in nanoparticles can overcome these limitations and can control/target their release under specific conditions. Therefore, nanotechnology provides an ideal carrier system to improve the pharmacokinetics and bioavailability of polyphenols.

Although nanoparticles are nearly perfect as carriers, their toxicity and side effects still need to be considered and minimized before clinical application. Because polyphenols are natural compounds that need to be taken for long periods of time to prevent and treat disease, it is important to understand the toxic side effects of nanoparticles when they accumulate in the body, especially if the nanoparticles have a low encapsulation rate. It is therefore necessary to establish standardized in vitro and in vivo models and conduct safety tests to promote the development and application of new nanoparticles beneficial to human health.

## Figures and Tables

**Table 1:** Antioxidant activities of some extracts/compounds from plants.

Polyphenols	Antioxidant Activity	Detection Method *	References
Extracts of Hippophae species	Regulate enzyme activity, affect the antioxidant reaction of cells	DPPH assay	[27,28]
Extracts of sweet potato leaves	Decrease the level of intracellular ROS	Photochemiluminescence assay, ORAC assay	[29,30]
Polyphenols from stevia rebaudiana	Radical scavenging, regulate enzyme activity	DPPH assay, ABTS assay	[31,32]
Curcumin	Direct reaction with free radicals, regulation of antioxidant-related enzyme activity and gene expression	DPPH assay, ABTS assay, total phenolic content assays	[33,34,35]
Extracts of Nymphaea nouchali leaf	Reducing DNA damage and attenuating oxidative stress-induced cell death	FRAP assay, ORAC assay, DPPH assay	[36]
Persimmon vinegar polyphenols	Activate of the Nrf2 antioxidative pathway	Fluorescent probe method, DPPH assay, total phenolic content assays	[37,38,39]
Anthocyanins	Radical scavenging, reduce the catalytic effect of metal ions	DPPH assay, T-AOC assay, ABTS assay, FRAP assay	[40,41,42]
Grape seed extract	Decrease the oxidized LDL in plasma, regulate enzyme activity	Antioxidant enzyme activity, DPPH assay, ORAC assay, ABTS assay	[43,44,45]
(−)-Epicatechin and procyanidin	Preservatives for fruit, radical scavenging	DPPH assay, hydroxyl radical scavenging capacity method, superoxide anion radical method	[46,47]
Extracts of blueberries	Regulate enzyme activity, chelate trace metals, regulate miRNA	FRAP assay, DPPH assay, ABTS assay	[48,49]
Extracts of pine	Radical scavenging, the skin against oxygen reactive species	DPPH assay, superoxide anion radical method, hydroxyl radical scavenging capacity method	[50,51]
Extracts of tea	Increase antioxidant enzyme activity, inhibit lipid peroxidation, radical scavenging	DPPH assay, FRAP assay, TEAC assay	[52,53]

* DPPH, 2,2-diphenyl-1-picrylhydrazyl; ORAC, oxygen radical absorbance capacity; ABTS, 2,2’-azino-bis(3-ethylbenzothiazoline-6-sulfonic acid; FRAP, fluorescence recovery after photobleaching; T-AOC, total antioxidant capacity; TEAC, trolox equivalent antioxidant capacity.

**Table 3:** Polyphenols complexed with phospholipids.

Phytosomal Formulations	Biological Activity	Route of Administration	Reference
Moringa oleifera leaf phytophospholipid complex	Wound healing	In vitro	[138]
Quercetin phytosome	Antimicrobial, anti-infammatory, anticancer	Oral	[139]
Curcumin phytosome	Antioxidant	In vitro, oral	[141,142]
Rutin-phospholipid	Anticytotoxicity, neuroprotection	In vitro	[143]
Catechin phyto-phospholipid	Antioxidant	In vitro	[144]
Luteolin phytosome	Hepatoprotective	Oral	[145]
Silybin phospholipid	Hepatoprotective, antioxidant, anticancer	In vivo	[140,146]
EGCG phytosome	Anticancer	Oral	[147]
Grape seed phytosome	Anticancer	Oral	[148]
Quercetin phytosome	Antioxidant, anti-inflammatory	Oral	[149]
Silybin phytosome	Hepatoprotective	Oral	[150]
Persimmon phytosome	Antioxidant	Oral	[151]
